# Broadband Enhancement of Cherenkov Radiation Using Dispersionless Plasmons

**DOI:** 10.1002/advs.202200538

**Published:** 2022-07-21

**Authors:** Hao Hu, Xiao Lin, Dongjue Liu, Hongsheng Chen, Baile Zhang, Yu Luo

**Affiliations:** ^1^ Interdisciplinary Center for Quantum Information State Key Laboratory of Modern Optical Instrumentation ZJU‐Hangzhou Global Science and Technology Innovation Center College of Information Science and Electronic Engineering Zhejiang University Hangzhou 310027 P. R. China; ^2^ School of Electrical and Electronic Engineering Nanyang Technological University 50 Nanyang Avenue Singapore 639798 Singapore; ^3^ International Joint Innovation Center ZJU‐UIUC Institute Zhejiang University Haining 314400 P. R. China; ^4^ Division of Physics and Applied Physics School of Physical and Mathematical Sciences Nanyang Technological University 21 Nanyang Link Singapore 637371 Singapore; ^5^ Centre for Disruptive Photonic Technologies Nanyang Technological University Singapore 637371 Singapore; ^6^ UMI 3288 CINTRA CNRS/NTU/THALES Nanyang Technological University 50 Nanyang Drive Singapore 637553 Singapore

**Keywords:** 2D materials, Cherenkov radiation, spatial nonlocality, surface plasmons

## Abstract

As one of leading technologies in detecting relativistic particles, Cherenkov radiation plays an essential role in modern high‐energy and particle physics. However, the limited photon yield in transparent dielectrics makes efficient Cherenkov radiation only possible with high‐energy particles (at least several MeV). This restriction hinders applications of Cherenkov radiation in free‐electron light source, bio‐imaging, medical therapy, etc. Broadband enhancement of Cherenkov radiation is highly desired for all these applications, but still widely acknowledged as a scientific challenge. To this end, a general approach is reported to enhance the photon yield of Cherenkov radiation using dispersionless plasmons. Broadband dispersionless plasmons can be realized by exploiting either the acoustic nature of terahertz plasmons in a graphene‐based heterostructure or the nonlocal property of optical plasmons in a metallodielectric structure. When coupled to moving electrons, such dispersionless plasmons give rise to a radiation enhancement rate more than two orders of magnitude (as compared with conventional Cherenkov radiation) over an ultrabroad frequency band. Moreover, since the phase velocity of dispersionless plasmons can be made as small as the Fermi velocity, giant radiation enhancements can be readily induced by ultralow‐energy free electrons (e.g., with a kinetic energy down to 3 eV), without resorting to relativistic particles.

## Graphical abstract

Surface plasmons are proposed to enhance Cherenkov radiation. However, the enhanced photon emission is generally accompanied with the narrow bandwidth. This work investigates a special type of surface waves, i.e., the dispersionless surface plasmon, that can enable broadband efficient Cherenkov radiation. This finding facilitates the application of Cherenkov radiation in highly compact free‐electron light sources.

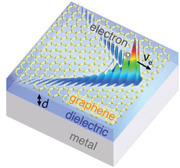

## 1 Introduction

Cherenkov radiation refers to the phenomenon where a swift charged particle emits light when it moves at a velocity larger than the phase velocity of light in the host medium.^[^ ^1^, ^2^ ^]^ After extensive explorations in the past decades, Cherenkov radiation has found enticing applications in high‐energy particle detectors and produced a profound influence on different realms, ranging from astrophysics to information and life science.^[^ ^3^, ^4^, ^5^, ^6^, ^7^, ^8^, ^9^, ^10^, ^11^, ^12^, ^13^, ^14^, ^15^ ^]^

Although Cherenkov radiation in the homogenous dielectric is broadband in nature, its applications to free‐electron radiation sources have been thus far restricted by the limited photon yield (e.g., at most 500 photons per centimeter in the visible band^[^ ^16^ ^]^). To generate sufficient photons for detection, traditional Cherenkov devices are generally bulky in size and oftentimes applied with high‐energy electrons. The problem of low photon efficiency is even more critical when the free charged particle interacts with low‐density materials. For instance, the ring‐imaging Cherenkov detectors (a typical high‐energy particle detector) require gas chambers of at least several meters in length and high‐energy‐particle incidence with the kinetic energy larger than 1 GeV.^[^ ^17^, ^18^, ^19^ ^]^ Such large chambers and high‐energy electrons are unsuitable for the implementation of on‐chip light sources. In fact, the limited photon yields also hinder many other promising applications of Cherenkov radiation, particularly in photodynamic therapy, which is now emerging as a modality for cancer therapy.^[^ ^20^, ^21^ ^]^ Such clinic applications normally require a large fluence rate of photons generated by low‐energy electrons, which is still challenging with state‐of‐the‐art Cherenkov light sources. Hence, enhancing the photon yield is highly desired for widening the applications of Cherenkov radiation.

To this end, surface plasmons (e.g., metal plasmons^[^ ^22^, ^23^, ^24^, ^25^ ^]^ and graphene plasmons^[^ ^26^, ^27^, ^28^ ^]^) have been proposed as a potential route to enhance the Cherenkov radiation yield. A large emission enhancement is obtained by coupling the free charged particles to deep‐subwavelength surface plasmons. However, the highly dispersive nature of conventional optical and terahertz plasmons makes the efficient enhancement of Cherenkov radiation only possible within a narrow frequency range.^[^ ^29^, ^30^, ^31^, ^32^, ^33^, ^34^ ^]^ Until now, how to enhance the photon yield of Cherenkov radiation without sacrificing the radiation bandwidth still remains an open question.

Here, we show that broadband enhancement of Cherenkov radiation can be achieved by coupling the free charged particle to dispersionless plasmons. The dispersionless plasmons refer to a special kind of surface electromagnetic modes which possess a nearly nondispersive propagation constant over a broad frequency band. They differ from conventional surface plasmon modes widely existing in photonic crystals, metamaterials/metasurface, 2D materials, etc. Such a novel mode can be made from acoustic plasmons supported by the graphene‐based heterostructures and metal plasmons supported by the nonlocal metallodielectric structure.^[^ ^35^, ^36^, ^37^, ^38^, ^39^, ^40^, ^41^ ^]^ For example, with judicious structural design, graphene plasmons behave as dispersionless acoustic plasmons. The achromatic nature of acoustic plasmons not only enhances the photon yield over an ultrabroadband frequency range (e.g., below 50 THz) but also makes the emission angle of Cherenkov photons robust against the frequency variation. Moreover, with proper engineering, dispersionless acoustic plasmons can propagate at a speed close to the Fermi velocity of the graphene (or metal), even considering the influence of nonlocality. Such a highly confined feature makes efficient photon generation possible even with ultralow‐energy electrons with a velocity down to *c*/300 (i.e., corresponding to kinetic energy of 3 eV), where *c* is the light speed in free space. We remark that this velocity is even smaller than the lowest experimentally reported value of *c*/32 (i.e., corresponding to kinetic energy of 250 eV) for Cherenkov radiation in hyperbolic metamaterials.^[^ ^42^ ^]^ In addition, the gate‐tunable plasmonic dispersion allows us to further engineer emission behaviors of Cherenkov radiation in a flexible way (see Sections S6–S8, Supporting Information). These unique advantages make dispersionless plasmons a powerful approach to design broadband free‐electron light sources at terahertz and X‐ray frequencies.

## 2 Results and Discussion

**Figure 1:**
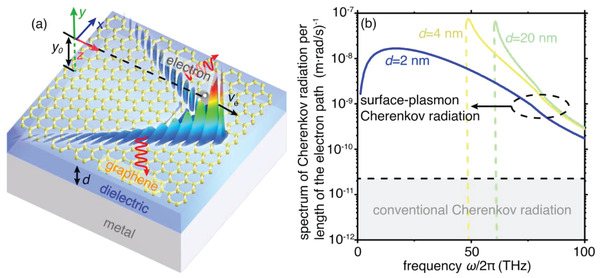
An surface‐plasmon route to broadband enhanced Cherenkov radiation with ultralow‐energy free electrons. a) Schematic of surface‐plasmon Cherenkov radiation. A free electron with a velocity of $\bar{v}=\hat{z}\;v_{e}$ travels in parallel to and close to the graphene sheet. The monolayer graphene is separated from the metal substrate by a dielectric spacer with a thickness of *d*. The red arrows correspond to the emission direction of surface‐plasmon Cherenkov radiation. Without particular statement, we use the chemical potential of *μ*_c_ = 0.3 eV, the relaxation time of *τ* = 0.5 ps for graphene; we adopt the silicon as the dielectric; and we set *v*_e_ = 1.3*v*_F_, where *v*_F_ = *c*/300 is the Fermi velocity in graphene. b) Spectrum of surface‐plasmon Cherenkov radiation per unit length of the electron path. The vertical dashed lines denote the cutoff frequencies of surface‐plasmon Cherenkov radiation. The black dashed line indicates the upper bound of the spectrum for conventional Cherenkov radiation induced by a swift electron moving inside any homogeneous dielectric.

For conceptual illustration, we consider a free electron moving at a velocity of ${\bar{v}}_{e}=\hat{z}\;v_{e}$ in the vacuum and atop a graphene‐based heterostructure, seeing the schematic in **Figure** **1**a. The trajectory of the free electron is parallel to the surface of the graphene‐based heterostructure. We set the vertical distance between the electron trajectory and graphene sheet as *y*_0_ = 5 nm to enhance the efficient elecromagnetic coupling between the free electron and surface plasmons. Notably, the electron beam is generally divergent in the practical implementation, potentially enhancing the probability of the collision between the free electrons and graphene‐based heterostructure. One option to reduce the divergence of the electron beam could be the application of an external magnetic field. The monolayer graphene is separated from the metal substrate by a dielectric spacer with a thickness of *d*. To make our study more realistic, we take into consideration the nonlocal effects in graphene and model the graphene conductivity by a spatially dispersive surface conductivity of *σ*_s_(*ω*,*q*) through the random phase approximation,^[^ ^43^, ^44^, ^45^ ^]^ where *ω* is the angular frequency and *q* is the in‐plane wavevector. Silicon and aluminium are chosen as the dielectric spacer and the metal substrate, respectively, with their permittivities extracted from experiments.^[^ ^46^, ^47^ ^]^ In the experimental fabrication, the adoption of silicon as the dielectric spacer generally presents challeges in the quick oxidization into SiO/SiO_2_ during the epitaxially grown process. To avoid this problem, the epitaxial growth of the silicon requires a vacuum environment. This graphene‐based heterostructure can support acoustic plasmons when the thickness of the dielectric spacer is decreased to several nanometers.^[^ ^37^, ^38^ ^]^

At such length scales, acoustic plasmons can enhance the photon yield of Cherenkov radiation over a broad frequency range. To demonstrate this point, we plot in Figure 1b the radiation spectrum of the free electron, where the separation *d* is set as 2, 4, and 20 nm, respectively (see the calculation details in Section S4, Supporting Information). For comparison, the black straight dashed line in Figure 1b gives the spectrum of conventional Cherenkov radiation induced by a free electron travelling in a homogeneous dielectric (e.g., silicon used in the calculation). In the conventional Cherenkov radiation process, the photon number generated per unit length of the electron path increases with the increasing product of particle velocity and refractive index of surrounding material. Particularly, when the product of particle velocity and refractive index goes to infinity, the photon yield in a homogenous dielectric approaches to its upper bound of 2.4 × 10^−11^ (m rad s^−1^)^–1^ (see the black dashed line in Figure 1b). In sharp contrast, the number of photons emitted in terms of acoustic plasmons in the structure with *d *= 2 nm is enhanced by at least two orders of magnitude within a broadband frequency below 50 THz (see the blue solid line in Figure 1b), even if the free electron moves at a relatively small velocity *v*_e_ = 1.3 *v*_F_ = *c*/230 (where the *v*_F_ = *c*/300 is the Fermi velocity of graphene). Such a broadband enhancement can hardly be achieved by conventional surface plasmons in the structure with *d* *>* 4 nm. In the experiment, the detection of acoustic‐plasmon Cherenkov radiation can be achieved with the silicon‐based blocked impurity band detectors and grating technology (see more discussions in Section S11, Supporting Information).

**Figure 2:**
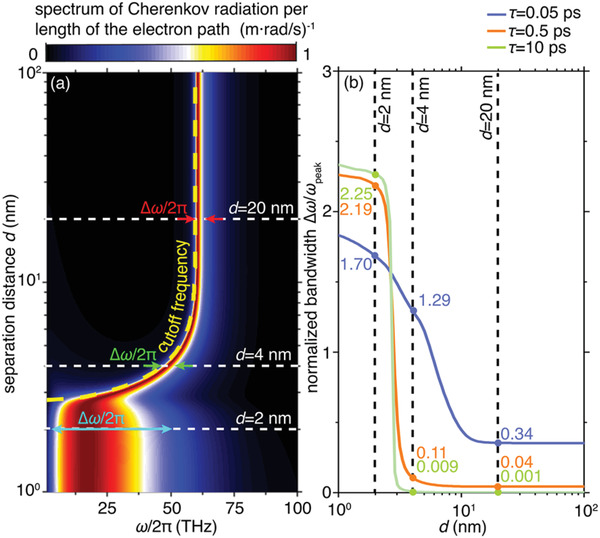
Influence of the separation distance on the bandwidth of efficient surface‐plasmon Cherenkov radiation. a) Spectrum of surface‐plasmon Cherenkov radiation per unit length of the electron path as a function of the separation and the frequency. For each separation, the spectrum is normalized by the maximum value, whose corresponding frequency is denoted as *ω*_peak_. The cutoff frequency of Cherenkov radiation is highlighted by the yellow dashed curve. b) Normalized bandwidth of efficient surface‐plasmon Cherenkov radiation versus the separation distance. To consider the influence of loss, different relaxation times *τ* for graphene are studied in (b).

Next, we quantatively demonstrate that the bandwidth of surface‐plasmon Cherenkov radiation can be flexibly engineered by the seperation *d* between the graphene sheet and the metal substrate. In particular, if *d* is decreased to 3 nm, the spectrum shows an abrupt increase in the bandwidth, accompanied by a sharp reduction in the cutoff frequency *ω*_c_, as shown in **Figure** **2**a. In order to quantify the sharp transition of the bandwidth, we plot the normalized bandwidth (namely Δ*ω*/*ω*_peak_) as a function of *d* in Figure 2b (where *ω*_peak_ is the frequency at which the peak radiation occurs). Apparently, the normalized bandwidth at small separations *d* ≤ 2 nm (i.e., Δ*ω*/*ω*_peak_ > 2) is over one order of magnitude larger than that at *d* ≥ 4 nm (i.e., Δ*ω*/*ω*_peak_ < 0.2). Moreover, the relaxation time is a key parameter that characterizes materials’ loss, thus influences the emission behaviors of Cherenkov radiation. Increasing (decreasing) the relaxation time makes such a bandwidth transition sharper (smoother) (Figure 2b). As a result, the contrast of bandwidths at *d *= 2 nm and *d *= 20 nm is changing from 5 to 225, when the relaxation time varies from 0.05 to 10 ps.

The sharp increase in the bandwidth is related to the emergence of broadband acoustic plasmons at the small separation *d*. At large separations, the heterostructure propagates conventional graphene plasmons with a dispersion relation $\omega \propto \sqrt{q}$ akin to that of deep water waves^[^ ^48^, ^49^, ^50^ ^]^ (see the case with *d* = 20 nm in Figure S2, Supporting Information). When the separation *d* decreases down to several nm, graphene plasmons become acoustic plasmons with a dispersion relation approximately satisfying *ω*∝*q*, analogous to that of shallow water waves^[^ ^48^ ^]^ (see the case with *d* = 2 nm in Figure S2, Supporting Information).

**Figure 3:**
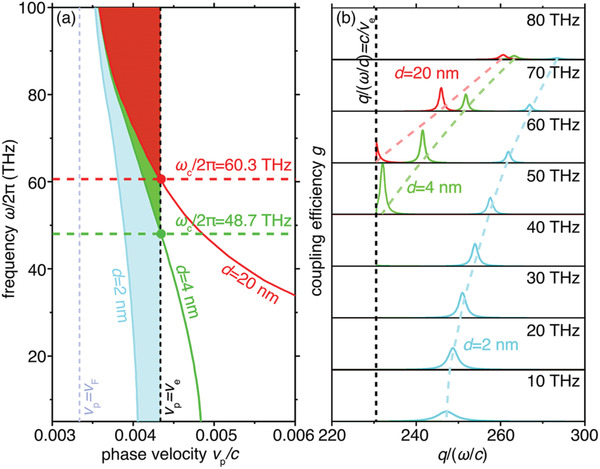
Influence of the separation distance on the phase velocity and electron‐plasmon coupling factor. a) Phase velocity of surface plasmons supported by the graphene‐based heterostructure as schematically shown in Figure 1a under different values of the separation distance. b) Electron‐plasmon coupling factor versus the in‐plane wavevector under different values of the separations. The coupling factors are in the same scale at all the studied frequencies. Other parameters setup here are: we set *v*_e_ = 1.3*v*_F_, where *v*_F_ = *c*/300.

The phase velocity of conventional surface plasmons generally increases with decreasing frequency and reaches a value comparable to *c* at extremely low frequencies. The large phase velocity gives rise to a lower bound frequency cutoff, preventing low‐energy electrons from emitting Cherenkov photons in the low frequency range (as Cherenkov radiation requires the incident electrons move faster than the phase veloclity of light in the host medium^[^ ^2^ ^]^). We remark on that the nearly nondispersive nature of acoustic plasmons offers a possible solution to this problem, enabling efficient Cherenkov radiation at extremely low frequencies even with ultraslow electrons. To illustrate this point, we plot in **Figure** **3**a the phase velocities of surface plasmons supported by the graphene‐based heterostructure at three typical separations, i.e., *d* =  2, 4, and 20 nm. Apparently, the phase velocity of conventional surface plasmons increases dramatically as the frequency decreases and reaches a maximum value of *c*/4 at low frequencies for *d* = 20 nm, whereas, acoustic plasmons supported by the heterostructure at *d* = 2 nm have a nearly constant phase velocity, which remains smaller than *c*/250 even at the zero frequency. Such a small nondispersive phase velocity eliminates the lower bound frequency cutoff of Cherenkov radiation even under low‐energy electron incidence. To demonstrate this point, we set electron velocity as *v*_e_ = *c*/230 (which is close to the Fermi velocity of graphene *v*_F_ = *c*/300). In this case, Cherenkov radiation is prohibited below 60.3 THz (48.7 THz) for *d* = 20 nm (*d* = 4 nm), while the broadband emission of acoustic plasmons is still allowed even at the zero frequency.

Moreover, the achromatic nature of acoustic plasmons makes the electron‐plasmon coupling factor robust against the frequency variation. The free electron generally couples to surface plasmons in a finite range of in‐plane wavevectors. The coupling factor between the free electron and surface plasmon is closely related to the normalized wavevector *q*/(*ω*/*c*) of excited surface plasmon (see the definition of coupling factor in Section S4, Supporting Information). Owing to the linear dispersion of acoustic plasmons, *q*/(*ω*/*c*) and hence, the coupling factor varies smoothly within a broad frequency range below 50 THz (see the cyan curves corresponding to *d* = 2 nm in Figure 3b). Such a smooth variation of coupling factor at *d *= 2 nm is responsible for the broadband enhancement of Cherenkov radiation. In sharp contrast, the highly dispersive nature of conventional surface plasmons makes *q*/(*ω*/*c*) extremely sensitive to the frequency variation (see green and red curves corresponding to *d* = 4 nm and *d* = 20 nm in Figure 3b). As a result, although the maximum coupling factor at *d *> 4 nm is larger than that at *d *= 2 nm, the strong dispersion makes the electron‐plasmon coupling significant only within a narrow frequency range (e.g., 50–70 THz for *d* = 4 nm and 60–70 THz for *d* = 20 nm). The significant frequency‐dependence of coupling factor in the case of *d *> 4 nm leads to the narrow bandwidth of conventional Cherenkov radiation.

**Figure 4:**
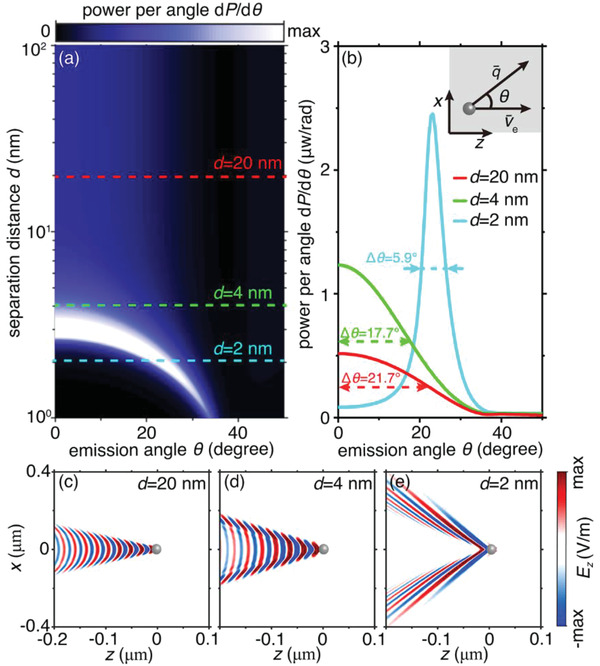
Influence of the separation distance on the angular spectrum of surface‐plasmon Cherenkov radiation in the time domain. a) Radiation power as a function of the emission angle and the separation distance. b) Radiation power of surface‐plasmon Cherenkov radiation as a function of the emission angle under different values of separation distance. c–e) Field distribution of excited surface‐plasmon Cherenkov radiation in the time domain. The electric field is plotted at the plane of *y* = − *y*_0_. At *d* = 20 nm (*d* = 4 nm), the free electron produces directionless plane‐like waves. In sharp contrast, at *d* = 2 nm, directional soliton‐like waves occur.

Remarkably, owing to the nondispersive feature of acoustic plasmons, Cherenkov radiation in our platform is highly directional. To demonstrate this point, we plot in **Figure** **4**a the time‐domain intensity of Cherenkov radiation as a function of the separation *d* and the emission angle *θ*, where *θ* represents the angle between the particle velocity ${\bar{v}}_{e}$ and phase velocity ${\bar{v}}_{p}$ of excited surface plasmons. Such an emission angle of Cherenkov radiation is generally dispersive as *θ* (*ω*) = cos ^−1^(*v*_p_(*ω*)/*v*_e_) , with its upper bound fixed at *θ*_max_ = cos ^−1^(*v*_F_/*v*_e_). Apparently, when *d* ≥ 4 nm, the lower bound of emission angle is 0° since *v*_p, min_ < *v*_e_. As a result, the conventional surface plasmons excited by the moving electron spread over a broad angular band (e.g., the half width at full maximum Δ*θ* = 21.7° (11.7°) for *d* = 20 nm (4 nm), as shown in Figure 4b) with the maxium intensity always centred at *θ* = 0°. The angular spectrum for *d* > 4 nm is phenomenally reflected as the directionless time‐domain near‐field radiation pattern with the dominance of plane‐like waves propating at *θ* = 0° (Figure 4c,d). On the contrary, when *d* is reduced below 3 nm, the lower bound of emission angle is nonzero but determined by *θ*_min_ = cos ^−1^(*v*_p, min_/*v*_e_) with *v*_p, min_ > *v*_e_. Therefore, a sharp decrease in Δ*θ* (e.g., Δ*θ* = 5.9° for *d* = 2 nm) is observed, with the maximum radiation intensity shifted to the angle within a narrower band from *θ*_min_ to *θ*_max_ (Figure S6, Supporting Information). The decrease in Δ*θ* corresponds to an enhancement of the emission directivity. Consequently, the peak radiation intensity at *d* = 2 nm is about five times larger than that at *d* = 20 nm, as shown in Figure 4b. The enhanced emission directionality at *d* = 2 is responsible for the disappearance of directionless plane‐like waves but the emergence of directional soliton‐like waves as plotted in Figure 4e.

**Figure 5:**
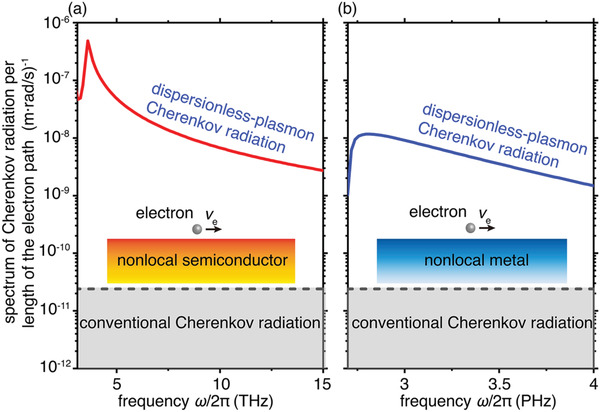
Dispersionless‐plasmon Cherenkov radiation in semiconductor/metal considering the nonlocality. a) Spectrum of dispersionless‐plasmon Cherenkov radiation per unit length of the electron path on nonlocal semiconductor substrate. In calculation, we choose InSb as the semiconductor. Parameters adopted for indium antimonide (InSb) are: the permittivity for bound charges *ε*_∞_ = 15.6, the plasmonic frequency *ω*_p_/2*π* = 7.16 × 10^13^ THz and the damping rate *γ* = 0.01*ω*_p_. b) Spectrum of dispersionless‐plasmon Cherenkov radiation per unit length of the electron path on nonlocal metal substrate. In calculation, we choose silver (Ag) as the metal. Parameters adopted for the Ag are: the permittivity for bound charges *ε*_∞_ = 1, the plasmonic frequency *ω*_p_ = 1.37 × 10^16^ rad s^−1^ and the damping rate *γ* = 0.01*ω*_p_. The black dashed line indicates the upper bound of the spectrum for conventional Cherenkov radiation induced by a swift electron moving inside any homogeneous dielectric. Other parameters setup here are: *v*_e_ = 1.3*v*_F_ and *v*_F_ = *c*/300.

Finally, we highlight that our findings on the broadband enhancement of Cherenkov radiation are general as the dispersionless plasmons widely exist in many configurations in addition to the graphene‐based heterostructure. Semiconductors such as indium antimonide (InSb) and gallium arsenide (GaAs) are known to support surface plasmons owing to their high charge carrier density at room temperature.^[^ ^51^, ^52^ ^]^ In all these materials, the nonlocality from charge‐level interactions is non‐negligible above the bulk plasma frequencies, forcing the velocity of surface plasmons to approach the nonlocal parameter $\beta \;=\sqrt{3/5}\;v_{F}$ as the frequency increases (Figure S10, Supporting Information). As a result, the dispersion curve becomes nearly linear in high frequencies, enabling the broadband enhancement of Cherenkov radiation from low‐energy electrons. For example, the InSb considering the nonlocality can enhance Cherenkov radiation by two orders of magnitude in the frequency band from 3 to 15 THz (see **Figure** **5**a).^[^ ^53^, ^54^, ^55^ ^]^ Such a mechanism can also be extended to the metal plasmons such as silver (Ag),^[^ ^56^ ^]^ leading to the broadband efficient Cherenkov radiation in 2.7–4 PHz (i.e., 11–17 eV) in Figure 5b.

## 3 Conclusion

To conclude, we have demonstrated that the dispersionless plasmons provide a feasible route to enhance Cherenkov radiation in a broad frequency range. We emphasize that our work has far‐reaching implifications in following two aspects: First, our revealed sharp bandwidth transition of Cherenkov radiation is highly suitable for nanoscale sensing. Such a sharp transition is highly sensitive to the nearby environment changes, e.g., the separation variations in graphene‐based heterostructure (Figure S7, Supporting Information). Capturing the sharp transition by tuning the chemical potential of graphene thus offers a potential route to measure separations down to several nm. Second, our findings can apply to the neighbouring fields of surface plasmons such as the surface photonic poloritons. Surface photonic poloritons widely exist in polar crystals such as molybdenum trioxide (MoO_3_) and hexagonal boron nitride (hBN).^[^ ^57^ ^]^ A recent work has shown that a monolayer of polar material located at a few nanometers distance above a metal substrate can support dispersionless photonic polaritons,^[^ ^58^ ^]^ providing an enticing platform for broadband efficient Cherenkov radiation in mid‐infrared (mid‐IR) frequencies. Our findings thus facilitate the applications of Cherenkov radiation in highly desired THz/mid‐IR/X‐ray free‐electron radiation sources and novel‐type Cherenkov radiation based photodynamic therapy.

## 4 Methods

### 4.1 Dispersion Relation of Graphene Plasmons in Graphene‐Based Heterostructure

The graphene‐based heterostructure shown in Figure 1a is divided into three regions, i.e., region 1 (*y* > 0), region 2 (0 > *y *> −*d*) and region 3 (*y*<−*d*), where the monolayer graphene is located at *y *= 0 and the relative permittivity in region *j* is denoted as *ε*_ri_. According to the electromagnetic theory,^[^ ^48^ ^]^ the transverse magnetic (TM) fields in each region take the form as

(1) $$E_{\rho }\left(\bar{r},\omega \right)=\left\{\begin{array}{cc} \frac{1}{\omega \varepsilon_{0}}\frac{k_{y1}}{\varepsilon_{r1}}R_{p1}e^{ik_{y1}y}e^{iq\rho }y & >0\\ \frac{1}{\omega \varepsilon_{0}}\frac{k_{y2}}{\varepsilon_{r2}}\left(-T_{p2}e^{-ik_{y2}y}+R_{p2}e^{ik_{y2}y}\right)e^{iq\rho } & -d<y<0\\ -\frac{1}{\omega \varepsilon_{0}}\frac{k_{y3}}{\varepsilon_{r3}}T_{p3}e^{-ik_{y3}y}e^{iq\rho } & -d>y \end{array}\right.$$

(2) $$E_{y}\left(\bar{r},\omega \right)=\left\{\begin{array}{cc} -\frac{q}{\omega \varepsilon_{0}}\frac{1}{\varepsilon_{r1}}R_{p1}e^{ik_{y1}y}e^{iq\rho } & y>0\\ -\frac{q}{\omega \varepsilon_{0}}\frac{1}{\varepsilon_{r2}}\left(T_{p2}e^{-ik_{y2}y}+R_{p2}e^{ik_{y2}y}\right)e^{iq\rho } & 0>y>-d\\ -\frac{q}{\omega \varepsilon_{0}}\frac{1}{\varepsilon_{r3}}T_{p3}e^{-ik_{y3}y}e^{iq\rho } & -d>y \end{array}\right.$$

(3) $$H_{h}\left(\bar{r},\omega \right)=\left\{\begin{array}{cc} R_{p1}e^{ik_{y1}y}e^{iq\rho } & y>0\\ \left(T_{p2}e^{-ik_{y2}y}+R_{p2}e^{ik_{y2}y}\right)e^{iq\rho } & 0>y>-d\\ T_{p3}e^{-ik_{y3}y}e^{iq\rho } & -d>y \end{array}\right.$$

In the above equations, $\bar{q}=\;q\;\hat{\rho }={\bar{k}}_{x}\;+{\bar{k}}_{z}$ is the in‐plane wavevector, $k_{yi}=\sqrt{\varepsilon_{\mathrm{ri}}k_{0}^{2}-q^{2}}\;$( $k_{z}=\frac{\omega }{v_{e}}\;$) is the *y* (*z*) component of wavevector in the region *j*, and *T*_pj_ (*R*_pj_) is the transmission (reflection) coefficient in the region *j*.

By enforcing the boundary conditions, one can obtain the dispersion of TM graphene plasmons in the heterostructure, i.e.,

(4) $$\begin{array}{ccc} & & \left[\frac{\varepsilon_{r1}}{k_{y1}}+\frac{\varepsilon_{r2}}{k_{y2}}+\frac{\sigma_{s}\left(q,\omega \right)}{\omega \varepsilon_{0}}\right]\left(\frac{\varepsilon_{r2}}{k_{y2}}+\frac{\varepsilon_{r3}}{k_{y3}}\right)e^{-ik_{y2}d}\\ & & \;+\;\left[\frac{\varepsilon_{r1}}{k_{y1}}-\frac{\varepsilon_{r2}}{k_{y2}}+\frac{\sigma_{s}\left(q,\omega \right)}{\omega \varepsilon_{0}}\right]\left(\frac{\varepsilon_{r2}}{k_{y2}}-\frac{\varepsilon_{r3}}{k_{y3}}\right)\;e^{ik_{y2}d}=0 \end{array}$$

where the nonlocal conductivity of graphene *σ*_s_(*q*,*ω*) is given by method of random phase approximation (see Section S1, Supporting Information).

### 4.2 Radiation Pattern of Cherenkov Radiation in the Graphene‐Based Heterostructure

In the structural schematic shown in Figure 1a, the current density of a free electron $J\;\left(\bar{r},t\right)=\hat{z}\;v_{e}e\delta \left(x\right)\delta \left(y-y_{0}\right)\delta \left(z-v_{e}t\right)$ in the free space (with *ε*_r1_ = 1) induces a vector potential as^[^ ^9^, ^60^ ^]^

(5) $${\bar{\phi }}_{0}=\hat{z}\;\int_{-\infty }^{+\infty }dk_{x}\frac{ie}{8\pi^{2}k_{y1}}e^{ik_{x}x+ik_{y}\left|y-y_{0}\right|+i\frac{\omega }{v_{e}}z}$$

where *e* is the elementary charge. The vector potential enables to determine the source field of the free electron as

(6) $$\left\{\begin{array}{c} \bar{E}\left(\bar{r},\omega \right)=\frac{i}{\omega \varepsilon_{0}}\nabla \times \nabla \times {\bar{\phi }}_{0}\\ \bar{H}\left(\bar{r},\omega \right)=\nabla \times {\bar{\phi }}_{0} \end{array}\right.$$

Matching boundary conditions for the source fields leads to the induced fields, where the *z*‐component electric field is expressed as

(7) Ezr¯,t=2Ree8π2ve2ε0∫0∞dω∫−∞+∞dkxωky1q2Rp1eikxx+iωvez−iωt

Here *R*_*p*1_ is the reflection coefficient in the region 1 (see its analytical expression in Section S3, Supporting Information). Note that transverse electric radiation field is not considered due to their negligible contributions to surface‐plasmon Cherenkov radiation.

### 4.3 Spectrum of Cherenkov Radiation in the Graphene‐Based Heterostructure

Without loss of generality, the number of photons generated per unit frequency can be calculated via the Green function as^[^ ^60^ ^]^

(8) Γω=−4αc∫dz∫dz′ImGzz,indr¯,r¯′e−iωvez−z′

where $\alpha \;=\frac{e^{2}}{4\pi \varepsilon_{0}\hbar c}\;$is the fine structure constant and $G_{zz,\mathrm{ind}}\left(\bar{r},{\bar{r}}^{\prime }\right)$ is the *zz* component of induced dyadic Green function (see the analytical expression in Section S4, Supporting Information). The spectrum of Cherenkov radiation per unit length of electron path is thus given by

(9) ddLΓω=2απc∫ω/ve∞dqω/veq2Imk02−q2k021−ω/veq2Im−Rp1e2iky1y0

In particular, the coupling factor between the free electron and surface plasmons at a specific propagation constant *q* is determined by the term

(10) g=Im−Rp1e2iky1y0

## Conflict of Interest

The authors declare no conflict of interest.

## Supporting information

Supporting Information

Supporting Information

Supporting Information

Supporting Information

## Data Availability

The data that support the findings of this study are available in the Supporting Information of this article.
