# Supplementary material for: Broadband Enhancement of Cherenkov Radiation Using Dispersionless Plasmons
Source: Adv Sci (Weinh). 2022 Jul 21;9(26):2200538. doi: 10.1002/advs.202200538 (PMC9475543; doi:10.1002/advs.202200538)
Supplement: Supplementary file 1 — Supporting Information [file ADVS-9-2200538-s004.pdf]

ADVANCED SCIENCE
Open Access

Supporting Information

for *Adv. Sci.*, DOI 10.1002/advs.202200538

Broadband Enhancement of Cherenkov Radiation Using Dispersionless Plasmons

Hao Hu, Xiao Lin, Dongjue Liu, Hongsheng Chen, Baile Zhang* and Yu Luo**

Supplementary Materials for

"Broadband enhancement of Cherenkov radiation using dispersionless plasmons"

Hao Hu, Xiao Lin , Dongjue Liu, Hongsheng Chen, Baile Zhang and Yu Luo

Supplementary Material Guide:

--Section S1. Graphene's surface conductivity with the consideration of nonlocality.

--Section S2. Dispersion curve of surface plasmons in graphene-based heterostructures.

--Section S3. Calculation of the radiation pattern of Cherenkov radiation in the time domain.

--Section S4. Calculation of the frequency spectrum of Cherenkov radiation.

--Section S5. Calculation of the angular spectrum of Cherenkov radiation.

--Section S6. More discussions on the cut-off frequency of Cherenkov radiation.

--Section S7. More discussions on the frequency spectrum of Cherenkov radiation.

--Section S8. More discussions on the angular spectrum of Cherenkov radiation.

--Section S9. Measuring nanoscale distance via sharp bandwidth transition of Cherenkov radiation.

--Section S10. Dispersionless-plasmon Cherenkov radiation on metal/semiconductor substrate

--Section S11. Detection scheme of acoustic-plasmon Cherenkov radiation.

Section S1. Graphene's surface conductivity with the consideration of nonlocality

We adopt the random phase approximation (RPA) method to analytically model the graphene's surface conductivity with the consideration of nonlocality. The nonlocal surface conductivity is expressed as [1,2]

$$\sigma_{\mathrm{s}}(q,\omega) = -\mathrm{i}\omega\chi_{\tau}(q,\omega) \tag{1.1}$$

where q is the in-plane wavevector and ω is the angular frequency. In Eq. (1.1), the nonlocal susceptibility is given by

$$\chi_{\tau}(q,\omega) = \frac{\left(1+\frac{\mathrm{i}}{\omega\tau}\right)\chi\left(q,\omega+\frac{\mathrm{i}}{\tau}\right)}{1+\frac{\frac{\mathrm{i}}{\omega\tau}\chi\left(q,\omega+\frac{\mathrm{i}}{\tau}\right)}{\chi(\overline{q},0)}} \tag{1.2}$$

where τ is the relaxation time of carriers in graphene. Considering the low-temperature limit, the $\chi(\overline{q},\omega)$ can be analytically written as

$$\chi(q,\omega) = \frac{e^2}{4\pi\hbar}\left[\frac{8k_{\mathrm{F}}}{q^2 v_{\mathrm{F}}} + \frac{(G(-\Delta_-)-\mathrm{i}\pi)\Theta(-\Delta_- -1)+G(\Delta_-)\Theta(\Delta_- +1)-(G(\Delta_+)-\mathrm{i}\pi)}{\sqrt{\omega^2-q^2v_{\mathrm{F}}^2}}\right] \tag{1.3}$$

where $k_{\mathrm{F}} = \frac{\omega}{v_{\mathrm{F}}}$, $\Delta_{\pm} = \frac{\hbar\omega\pm 2\mu_{\mathrm{c}}}{\hbar q v_{\mathrm{F}}}$, $G(x) = x\sqrt{x^2-1} - \ln\left(x+\sqrt{x^2-1}\right)$ and Θ is the heaviside step function. In addtion, v_{F} is the Fermi velocity and μ_{c} is the chemical potential in graphene. When $|x| < 1$ ($|x| > 1$), the square root in $G(x)$ is chosen to yield a positive imaginary (real) part. Besides, the imaginary part of the logarithm should fall within $\left(-\pi,\ \pi\right)$. On the other hand, the analytical expression of $\chi(q,0)$ is obtained as

$$\chi(q,0) = \frac{e^2}{2\pi\hbar v_{\mathrm{F}} q}\left\{\frac{4k_{\mathrm{F}}}{q} - \Theta\left(1-\frac{2k_{\mathrm{F}}}{q}\right)\left[\frac{2k_{\mathrm{F}}}{q}\sqrt{1-\left(\frac{2k_{\mathrm{F}}}{q}\right)^2} - \cos^{-1}\frac{2k_{\mathrm{F}}}{q}\right]\right\}. \tag{1.4}$$

Section S2. Dispersion curve of surface plasmons in graphene-based heterostructures

We consider the graphene-based heterostructure as shown in Figure S1. To determine the dispersion curve of eigenmode (i.e., graphene plasmons) in this structure, we establish the (h, y, ρ) coordinate where the in-plane wavevector $\bar{q}$ of the eigenmode is parallel to the ρ-axis. In this coordinate system, the eigenmode (i.e., graphene plasmons) takes the form as

$$E_\rho(\bar{r},\omega) = \begin{cases} \frac{1}{\omega\varepsilon_0}\frac{k_{y1}}{\varepsilon_{r1}} R_{p1} e^{\mathrm{i}k_{y1}y}\, e^{\mathrm{i}q\rho} & y > 0 \\ \frac{1}{\omega\varepsilon_0}\frac{k_{y2}}{\varepsilon_{r2}} \left(-T_{p2} e^{-\mathrm{i}k_{y2}y} + R_{p2} e^{\mathrm{i}k_{y2}y}\right) e^{\mathrm{i}q\rho} & -d < y < 0 \\ -\frac{1}{\omega\varepsilon_0}\frac{k_{y3}}{\varepsilon_{r3}} T_{p3} e^{-\mathrm{i}k_{y3}y} e^{\mathrm{i}q\rho} & -d > y \end{cases} \qquad (2.1a)$$

$$E_y(\bar{r},\omega) = \begin{cases} -\frac{q}{\omega\varepsilon_0}\frac{1}{\varepsilon_{r1}} R_{p1} e^{\mathrm{i}k_{y1}y}\, e^{\mathrm{i}q\rho}, & y > 0 \\ -\frac{q}{\omega\varepsilon_0}\frac{1}{\varepsilon_{r2}} \left(T_{p2} e^{-\mathrm{i}k_{y2}y} + R_{p2} e^{\mathrm{i}k_{y2}y}\right) e^{\mathrm{i}q\rho} & 0 > y > -d \\ -\frac{q}{\omega\varepsilon_0}\frac{1}{\varepsilon_{r3}} T_{p3} e^{-\mathrm{i}k_{y3}y} e^{\mathrm{i}q\rho} & -d > y \end{cases} \qquad (2.1b)$$

$$H_h(\bar{r},\omega) = \begin{cases} R_{p1} e^{\mathrm{i}k_{y1}y}\, e^{\mathrm{i}q\rho}, & y > 0 \\ \left(T_{p2} e^{-\mathrm{i}k_{y2}y} + R_{p2} e^{\mathrm{i}k_{y2}y}\right) e^{\mathrm{i}q\rho} & 0 > y > -d \\ T_{p3} e^{-\mathrm{i}k_{y3}y} e^{\mathrm{i}q\rho} & -d > y \end{cases} \qquad (2.1c)$$

where $k_{yi} = \sqrt{\epsilon_{ri}k_0^2 - q^2}$ is the y component of wavevector in the region j and T_{pj} (R_{pj}) is the transmission (reflection) coefficient in the region j. Via applying the boundary condition on electromagnetic fields (i.e., tangent continuity of field components), we obtain the dispersion curve of graphene plasmon in the graphene-based heterostucture as,

$$\left[\frac{\varepsilon_{r1}}{k_{y1}} + \frac{\varepsilon_{r2}}{k_{y2}} + \frac{\sigma_s}{\omega\varepsilon_0}\right]\left(\frac{\varepsilon_{r2}}{k_{y2}} + \frac{\varepsilon_{r3}}{k_{y3}}\right) e^{-\mathrm{i}k_{y2}d} + \left[\frac{\varepsilon_{r1}}{k_{y1}} - \frac{\varepsilon_{r2}}{k_{y2}} + \frac{\sigma_s}{\omega\varepsilon_0}\right]\left(\frac{\varepsilon_{r2}}{k_{y2}} - \frac{\varepsilon_{r3}}{k_{y3}}\right) e^{\mathrm{i}k_{y2}d} = 0. \qquad (2.2)$$

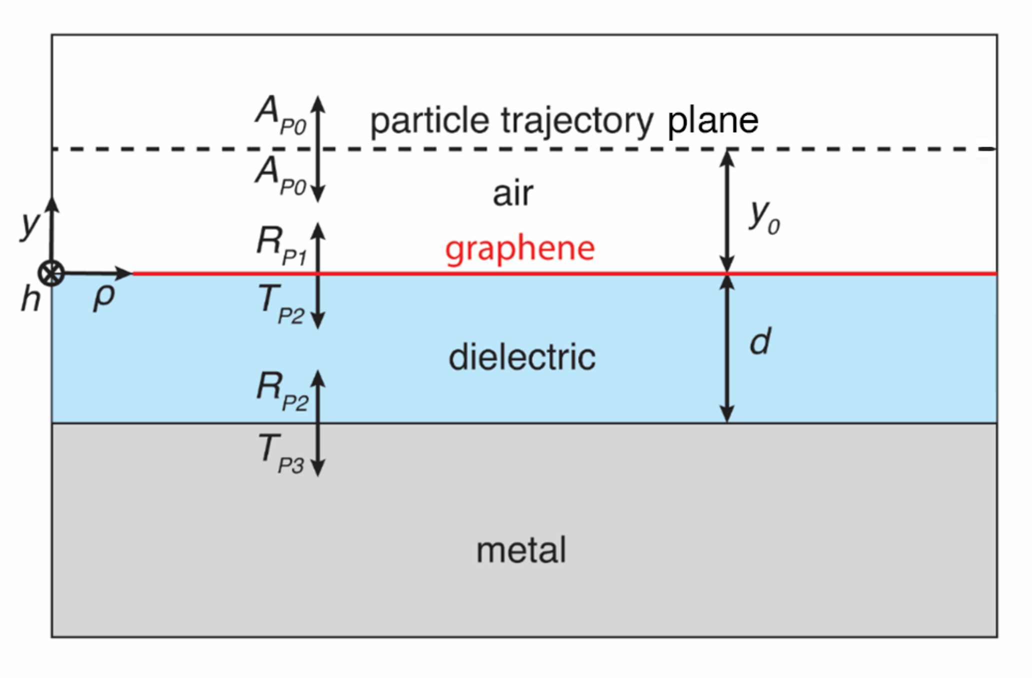

Figure S1. Structural schematic of the designed graphene-based heterostructure. Here, the distance between the particle trajectory plane and single-layer graphene sheet is y_0. The separation distance between the graphene and the metal is d. A_{p0} is the amplitude of TM-polarized source field. R_{pj} and T_{pj} are the reflection coefficients and transmission coefficients in the region j. The subscripts $j = 1,2,3$ refer to the air, dielectric and metal domain, respectively.

As a concrete example, we plot in Figure S2 the dispersion relation of surface plasmons supported by the graphene-based heterostructure as schematically shown in Figure 1a under different separation distances. The graphene-based heterostructure can support different kinds of surface plasmons by adjusting the separation distances. To be specific, when the separation distance is large enough (e.g. $d = 20$ nm), the heterostructure supports conventional plasmons, whose dispersion relation has $\omega \propto \sqrt{q}$ which is akin to that of deep water waves [3-5]. On the contrary, when the separation decreases to $d = 2$ nm, the surface mode supported by the heterostructure becomes dispersionless acoustic plasmons. Remarkably, the

dispersion relation of acoustic plasmons approximately has $\omega \propto q$, analogous to that of shallow water waves [3].

The acoustic plasmon results from the coupling between the graphene plasmon and its image in the metal substrate. When the coupling is strong enough, the surface mode is highly confined regardless of propagation constants. This makes graphene plasmons move extremely slowly (close to the Fermi velocity) in a broad frequency range. As a result, the dispersion curve is almost linear leading to the dispersionless acoustic plasmons.

We emphasize that such a linear dispersion relation of acoustic plasmons plays a key role to eliminate the chromatic dispersion, resulting in efficient Cherenkov radiation over a broad frequency range.

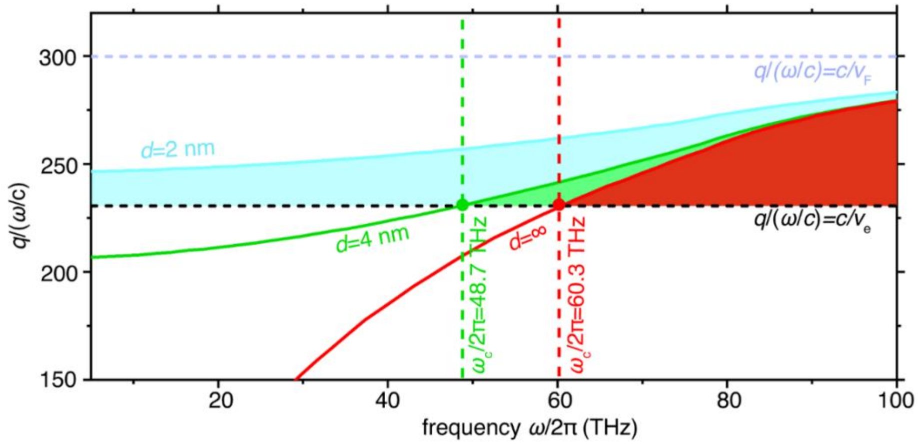

Figure S2. Dispersion relation of surface plasmons supported by the graphene-based heterostructure as schematically shown in Figure 1a under different values of the separations. The studied separation is 2 nm, 4 nm and ∞, respectively. Other parameter setups are same as that in Figure 1a.

Section S3. Calculation of the radiation pattern of Cherenkov radiation in the time domain

In the (x,y,z) coordinate as shown in Figure 1a, the current density of a free electron $J(\overline{r},t) = \hat{z}v_{\mathrm{e}}e\delta(x)\delta(y-y_0)\delta(z-v_{\mathrm{e}}t)$ induces a vector potential as [6]

$$\overline{\phi}_0 = \hat{z}\int_{-\infty}^{+\infty}\mathrm{d}k_x \frac{\mathrm{i}e}{8\pi^2 k_{y1}} e^{\mathrm{i}k_x x + \mathrm{i}k_y|y-y_0| + \mathrm{i}\frac{\omega}{v_{\mathrm{e}}}z} \ . \tag{3.1}$$

where e is the elementary charge. The source field is determined by the vector potential as

$$\begin{cases} \overline{E}(\overline{r},\omega) = \frac{\mathrm{i}}{\omega\varepsilon_0\varepsilon_{\mathrm{r}1}}\nabla\times\nabla\times\overline{\phi}_0 \\ \overline{H}(\overline{r},\omega) = \nabla\times\overline{\phi}_0 \end{cases} . \tag{3.2}$$

Since the graphene plasmons are transverse magnetic (TM) polarized, only TM-polarized incidence from the free electron interacts with the graphene plasmons. For convenience, we express the field component of TM-polarized light from the free electron in the (h,y,ρ) coordinate by using the rotation matrix

$$\begin{bmatrix}\hat{\rho}\\ \hat{h}\end{bmatrix} = \begin{bmatrix}\cos\alpha & -\sin\alpha \\ \sin\alpha & \cos\alpha\end{bmatrix}\begin{bmatrix}\hat{z}\\ \hat{x}\end{bmatrix} \tag{3.3}$$

where $\tan\alpha = \frac{k_x}{k_z}$. Thus, we determine the TM-polarized component of source field with a specific k_x in the (h,y,ρ) coordinate as,

$$\begin{cases} E_\rho^s = \frac{1}{\omega\varepsilon_0}A_{\mathrm{p}0}\left(-\frac{k_{y1}}{\varepsilon_{\mathrm{r}1}}\right)e^{\mathrm{i}k_{y1}|y-y_0|+\mathrm{i}q\rho} \\ E_y^s = \frac{1}{\omega\varepsilon_0\varepsilon_{\mathrm{r}1}}A_{\mathrm{p}0}(q\,\mathrm{sgn}(y-y_0))e^{\mathrm{i}k_{y1}|y-y_0|+\mathrm{i}q\rho} \\ H_h^s = A_{\mathrm{p}0}(-\mathrm{sgn}(y-y_0))e^{\mathrm{i}k_{y1}|y-y_0|+\mathrm{i}q\rho} \end{cases} \tag{3.4}$$

where $A_{\mathrm{p}0} = \frac{e\omega}{8\pi^2 q v_{\mathrm{e}}}$. Via matching the boundary conditions, the scattering coefficients of the induced scattering field are obtained as

$$R_{\mathrm{p}1} = \frac{m_1+m_2}{s_1+s_2} \tag{3.5a}$$

$$T_{\mathrm{p}2} = \frac{m_3}{s_1+s_2} \tag{3.5b}$$

$$R_{\mathrm{p2}} = \frac{m_4}{s_1+s_2} \tag{3.5c}$$

$$T_{\mathrm{p3}} = \frac{m_5}{s_1+s_2} \tag{3.5d}$$

where

$$m_1 = \left[\frac{k_{y1}}{\varepsilon_{\mathrm{r1}}} + \frac{k_{y2}}{\varepsilon_{\mathrm{r2}}}\left(-1 + \frac{\sigma_s k_{y1}}{\omega\varepsilon_0\varepsilon_{\mathrm{r1}}}\right)\right]\left(\frac{k_{y2}}{\varepsilon_{\mathrm{r2}}} + \frac{k_{y3}}{\varepsilon_{\mathrm{r3}}}\right)e^{-\mathrm{i}k_{y2}d}$$

$$m_2 = \left[\frac{k_{y1}}{\varepsilon_{\mathrm{r1}}} - \frac{k_{y2}}{\varepsilon_{\mathrm{r2}}}\left(-1 + \frac{\sigma_s k_{y1}}{\omega\varepsilon_0\varepsilon_{\mathrm{r1}}}\right)\right]\left(\frac{k_{y2}}{\varepsilon_{\mathrm{r2}}} - \frac{k_{y3}}{\varepsilon_{\mathrm{r3}}}\right)e^{\mathrm{i}k_{y2}d}$$

$$m_3 = 2\frac{k_{y1}}{\varepsilon_{\mathrm{r1}}}\left(\frac{k_{y2}}{\varepsilon_{\mathrm{r2}}} + \frac{k_{y3}}{\varepsilon_{\mathrm{r3}}}\right)e^{-\mathrm{i}k_{y2}d}$$

$$m_4 = 2\frac{k_{y1}}{\varepsilon_{\mathrm{r1}}}\left(\frac{k_{y2}}{\varepsilon_{\mathrm{r2}}} - \frac{k_{y3}}{\varepsilon_{\mathrm{r3}}}\right)e^{-\mathrm{i}k_{y2}d}$$

$$m_5 = 4\frac{k_{y1}}{\varepsilon_{\mathrm{r1}}}\frac{k_{y2}}{\varepsilon_{\mathrm{r2}}}e^{-\mathrm{i}k_{y2}d}$$

$$s_1 = \left[\frac{k_{y1}}{\varepsilon_{\mathrm{r1}}} + \frac{k_{y2}}{\varepsilon_{\mathrm{r2}}}\left(1 + \frac{\sigma_s k_{y1}}{\omega\varepsilon_0\varepsilon_{\mathrm{r1}}}\right)\right]\left(\frac{k_{y2}}{\varepsilon_{\mathrm{r2}}} + \frac{k_{y3}}{\varepsilon_{\mathrm{r3}}}\right)e^{-\mathrm{i}k_{y2}d}$$

$$s_2 = \left[\frac{k_{y1}}{\varepsilon_{\mathrm{r1}}} - \frac{k_{y2}}{\varepsilon_{\mathrm{r2}}}\left(1 + \frac{\sigma_s k_{y1}}{\omega\varepsilon_0\varepsilon_{\mathrm{r1}}}\right)\right]\left(\frac{k_{y2}}{\varepsilon_{\mathrm{r2}}} - \frac{k_{y3}}{\varepsilon_{\mathrm{r3}}}\right)e^{\mathrm{i}k_{y2}d}.$$

Finally, the time-domain radiation pattern of Cherenkov radiation from the moving electron in the graphene-based heterostructure is obtained by applying the reversed rotation matrix on the field components. For example, the field distribution E_z in region 1 (i.e., the air domain) is determined as

$$E_z(\overline{r},t) = 2\,\mathrm{Re}\left(\frac{1}{\omega\varepsilon_0}\int_0^{\infty}\mathrm{d}\omega\int_{-\infty}^{+\infty}\mathrm{d}k_x\left[-\frac{k_{y1}}{\varepsilon_{\mathrm{r1}}} + \frac{k_{y1}}{\varepsilon_{\mathrm{r1}}}R_{p1}\right]\frac{\omega}{v_{\mathrm{e}}q}A_{\mathrm{p0}}\right)e^{\mathrm{i}k_x x+\mathrm{i}\frac{\omega}{v_{\mathrm{e}}}z-\mathrm{i}\omega t}. \tag{3.6}$$

Section S4. Calculation of the frequency spectrum of Cherenkov radiation

We proceed to calculate the loss probability spectrum. The loss probability corresponds to the number of photons generated per unit length of electron path per unit frequency. The loss probability can be obtained via the Green function as [7]

$$\Gamma(\omega)=-\frac{4\alpha}{c}\int \mathrm{d}z\int \mathrm{d}z'\,\mathrm{Im}\left[G_{zz,\mathrm{ind}}(\bar{r},\bar{r}')e^{-\mathrm{i}\frac{\omega}{v_e}(z-z')}\right] \tag{4.1}$$

, where $\alpha=\frac{e^2}{4\pi\varepsilon_0\hbar c}$ is the fine structure constant. The *zz* component of induced dyadic Green function is

$$G_{\mathrm{zz,ind}}(\bar{r},\bar{r}')=\frac{1}{8\pi^2}\int_0^{\infty}q\mathrm{d}q\int_0^{2\pi}\mathrm{d}\theta\left[\underbrace{(\sin^2\theta)R_{\mathrm{TE}}e^{\mathrm{i}k_{y1}y_0}}_{\substack{\text{TE}-\text{polarized}\\ h\text{-component}}}+\underbrace{\left(\frac{k_{y1}^2}{k^2}\cos^2\theta\right)R_{\mathrm{TM}}e^{\mathrm{i}k_{y1}y_0}}_{\substack{\text{TM}-\text{polarized}\\ \rho-\text{component}}}\right]\frac{e^{\mathrm{i}qx\sin\theta+\mathrm{i}qz\cos\theta+\mathrm{i}k_{y1}y}}{k_{y1}} \tag{4.2}$$

, where θ is the in-plane angle relative to *z*-axis and $k=\sqrt{\varepsilon_{\mathrm{r1}}}k_0$. Since the TE-polarized components of Cherenkov radiation is generally weak and they cannot be enhanced by the surface plasmons, it is reasonable to neglect the term of TE-polarized components. As such, the loss probability can be reduced as

$$\Gamma(\omega)=\frac{2\alpha L}{\pi c}\mathrm{Re}\int_0^{\infty}q\mathrm{d}q\int_0^{\frac{\pi}{2}}\mathrm{d}\theta\left[\frac{1}{k_y}\left(\frac{k_y^2}{k^2}\cos^2\theta\right)R_{\mathrm{TM}}e^{2\mathrm{i}k_{y1}y_0}\delta\left(q\cos\theta-\frac{\omega}{v_{\mathrm{e}}}\right)\right] \tag{4.3}$$

where reflection coefficient R_{TM} corresponds to the -R_{p1} in Eq. (3.5a). Using the identity

$$\delta\left(q\cos\theta-\frac{\omega}{v_{\mathrm{e}}}\right)=\frac{1}{q}\delta\left(\cos\theta-\frac{\omega}{qv_{\mathrm{e}}}\right) \tag{4.4}$$

the expression of loss probability of free electrons in the graphene-based heterostructure can be further simplified as

$$\Gamma(\omega)=\frac{2\alpha L}{\pi c}\mathrm{Re}\int_{\omega/v_{\mathrm{e}}}^{\infty}\mathrm{d}q\left(\frac{\left(\frac{\omega/v_{\mathrm{e}}}{q}\right)^2\sqrt{\varepsilon_{\mathrm{r1}}k_0^2-q^2}}{\varepsilon_{\mathrm{r1}}k_0^2\sqrt{1-\left(\frac{\omega/v_{\mathrm{e}}}{q}\right)^2}}\right)R_{\mathrm{TM}}e^{2\mathrm{i}k_yy_0}. \tag{4.5}$$

Therefore, the expression of loss probability per unit length of electron path is

$$\frac{\mathrm{d}}{\mathrm{d}L}\Gamma(\omega) = \frac{2\alpha}{\pi c}\int_{\omega/v_\mathrm{e}}^{\infty}\mathrm{d}q\left(\frac{\left(\frac{\omega/v_\mathrm{e}}{q}\right)^2 \mathrm{Im}\sqrt{\varepsilon_{r1}k_0^2-q^2}}{\varepsilon_{r1}k_0^2\sqrt{1-\left(\frac{\omega/v_\mathrm{e}}{q}\right)^2}}\right)\mathrm{Im}\left[R_\mathrm{TM}e^{2\mathrm{i}k_{y1}y_0}\right]. \quad (4.6)$$

Importantly, the coupling factor between the free electron and surface plasmon at a specific propagation constant q is given by the term [8]

$$g = \mathrm{Im}\left[R_\mathrm{TM}e^{2\mathrm{i}k_{y1}y_0}\right]. \quad (4.7)$$

These equations are derived by assuming that the structure is sufficiently large in the lateral dimension (i.e., *x*-*z* plane). However, our results are valid as long as the in-plane size of structure is larger than the propagation length of generated surface plasmons, e.g., 4×4 μm^2 for graphene acoustic plasmons if the relaxation time is τ=1 ps.

On the other hand, the expression of loss probability per unit length of electron path in a homogeneous dielectric with the permittivity of ε_r is

$$\frac{\mathrm{d}}{\mathrm{d}L}\Gamma(\omega) = \frac{\alpha}{c}\left[1-\frac{1}{\varepsilon_\mathrm{r}}\left(\frac{c}{v_\mathrm{e}}\right)^2\right]\Theta\left[1-\frac{1}{\varepsilon_\mathrm{r}}\left(\frac{c}{v_\mathrm{e}}\right)^2\right] = \frac{\alpha}{c}\left[1-\left(\frac{1}{n\beta}\right)^2\right]\Theta\left[1-\left(\frac{1}{n\beta}\right)^2\right] \quad (4.8)$$

, where $\Theta(t)$ is the Heaviside step function. Eq. (4.8) clearly shows that the photon yield of Cherenkov radiation in a homogenous dielectric highly depends on the product of the electron velocity β and the refractive index n of dielectric. In particular, when the product $n\beta$ goes to the infinity, the photon yield approaches to its upper bound, i.e., $a/c \approx 2.4\times10^{-11}(\mathrm{m}\cdot\mathrm{rad/s})^{-1}$.

<u>**Section S5. Calculation of the angular spectrum of Cherenkov radiation**</u>

To obtain the angular spectrum of Cherenkov radiation, we simplify the Eq. (4.3) alternatively with the identity

$$\delta\left(q\cos\theta-\frac{\omega}{v_{\mathrm{e}}}\right)=\frac{1}{|\cos\theta|}\delta\left(q-\frac{\omega}{v_{\mathrm{e}}\cos\theta}\right). \tag{5.1}$$

As a consequence, Eq. (5.1) is simplified as

$$\Gamma(\omega)=\frac{2\alpha L\omega}{\pi c v_{\mathrm{e}}}\int_0^{\frac{\pi}{2}}d\theta\left(\frac{\mathrm{Im}\sqrt{\varepsilon_{\mathrm{r1}}k_0^2-\left(\frac{\omega/v_{\mathrm{e}}}{\cos\theta}\right)^2}}{\varepsilon_{\mathrm{r1}}k_0^2}\right)\mathrm{Im}\left[R_{\mathrm{TM}}e^{2\mathrm{i}k_{y1}y_0}\right] \tag{5.2}$$

Note that Eq. (5.2) is equivalent to Eq. (4.5). Thus, the power emitted by the free electron in the time domain can be determined with the loss probability as

$$P=\int_0^{\infty}\mathrm{d}\omega\Gamma(\omega)\hbar\omega\frac{v_{\mathrm{e}}}{L}=\int_0^{\infty}\mathrm{d}\omega\frac{e^2\mu_0\omega^2}{2\pi^3}\int_0^{\frac{\pi}{2}}\mathrm{d}\theta\left(\frac{\mathrm{Im}\sqrt{\varepsilon_{\mathrm{r1}}k_0^2-\left(\frac{\omega/v_{\mathrm{e}}}{\cos\theta}\right)^2}}{\varepsilon_{\mathrm{r1}}k_0^2}\right)\mathrm{Im}\left[R_{\mathrm{TM}}e^{2\mathrm{i}k_{y1}y_0}\right] \tag{5.3}$$

Therefore, we obtain the radiation power per angle in the time domain as

$$\frac{\mathrm{d}P}{\mathrm{d}\theta}=\frac{e^2\mu_0}{2\pi^3}\int_0^{\infty}\omega^2\mathrm{d}\omega\left(\frac{\mathrm{Im}\sqrt{\varepsilon_{\mathrm{r1}}k_0^2-\left(\frac{\omega/v_{\mathrm{e}}}{\cos\theta}\right)^2}}{\varepsilon_{\mathrm{r1}}k_0^2}\right)\mathrm{Im}\left[R_{\mathrm{TM}}e^{2\mathrm{i}k_{y1}y_0}\right]. \tag{5.4}$$

Section S6. More discussions on the cutoff frequency of Cherenkov radiation

We proceed to analyze the influence of permittivity and chemical potential on the cutoff frequency of Cherenkov radiation. As shown Figure S3, the permittivity of dielectric spacer and the chemical potential in graphene provide new degrees of freedom to control the cutoff frequency of Cherenkov radiation, in addition to the separations. To be specific, increasing the permittivity of dielectric spacer will reduce the cutoff frequency, while increasing the chemical potential of graphene will increase the cutoff frequency.

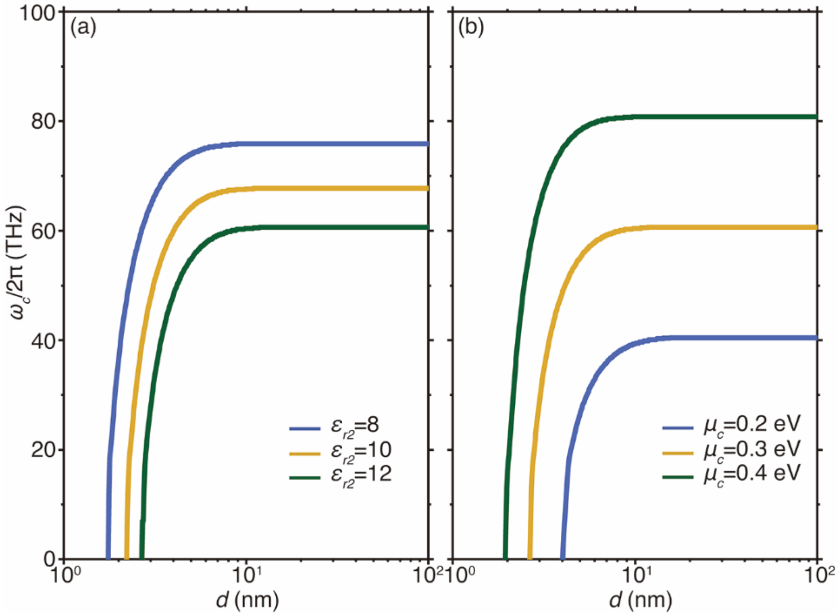

Figure S3. Controlling the cutoff frequency of Cherenkov radiation. a) The influence of the permittivity of dielectric spacer on the cutoff frequency. b) The influence of the chemical potential in graphene on the cutoff frequency. Other parameter setups are $v_{\mathrm{e}} = 1.3v_{\mathrm{F}}$ and $\tau = 0.5$ ps.

Section S7. More discussions on the frequency spectrum of Cherenkov radiation

In this section, we first discuss the influence of the permittivity of dielectric spacer and the chemical potential on the bandwidth of acoustic-plasmon Cherenkov radiation. From Figure S4, the increase of permittivity of dielectric spacer can effectively broaden the radiation bandwidth of acoustic-plasmon Cherenkov radiation, whereas there is an optimal value of chemical potential that enables the maximum bandwidth. Therefore, the permittivity of dielectric spacer and the chemical potential provide additional degrees of freedom to control the bandwidth of Cherenkov radiation in the proposed platform.

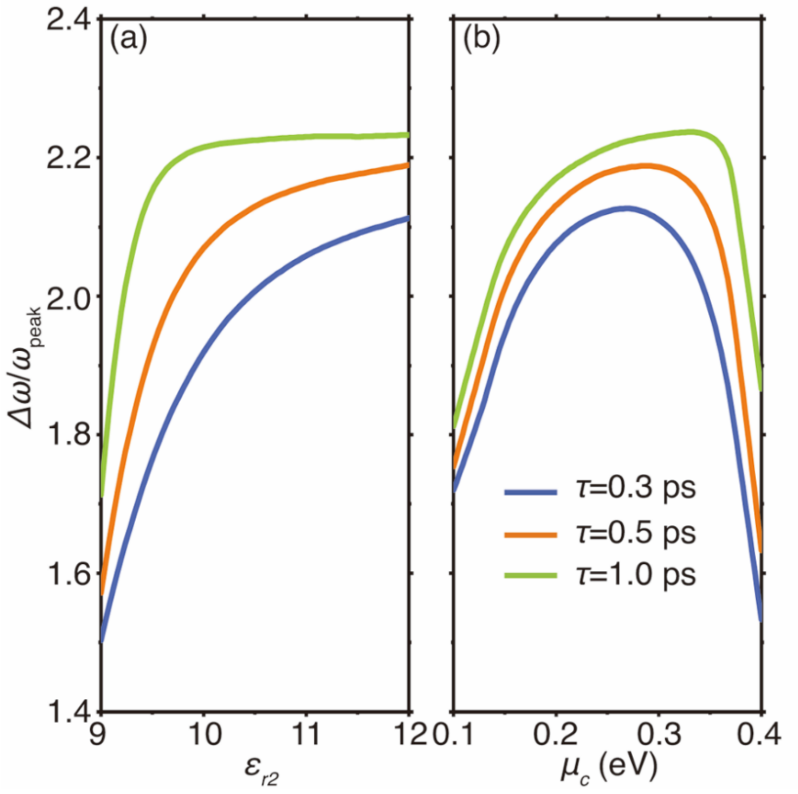

Figure S4. Controlling the frequency bandwidth of acoustic-plasmon Cherenkov radiation. a) Influence of the permittivity of dielectric spacer on the normalized bandwidth. The chemical potential is $\mu_c = 0.3$ eV. b) Influence of the chemical potential of graphene on the normalized bandwidth. The permittivity of dielectric is $\varepsilon_{r2} = 12$. In all the panels, the studied relaxation time are $\tau = 0.3$ ps, $\tau = 0.5$ ps and $\tau = 1.0$ ps, respectively. Other parameter setups are: $d = 2$ nm, $v_e = 1.3v_F$ and $v_F = 1 \times 10^6$ m/s.

Moreover, we explore the influence of the distance between the free electron and graphene sheet on the spectrum of surface-plasmon Cherenkov radiation with $d = 2$ nm. Figure S5 demonstrates that the dispersionless-plasmons still leads to the broadband enhancement of Cherenkov radiation below 50 THz, when y_0 varies from 5 nm to 15 nm. The flexibility in the distance between the free electron and graphene sheet facilitates the experiment realization of broadband efficient Cherenkov radiation.

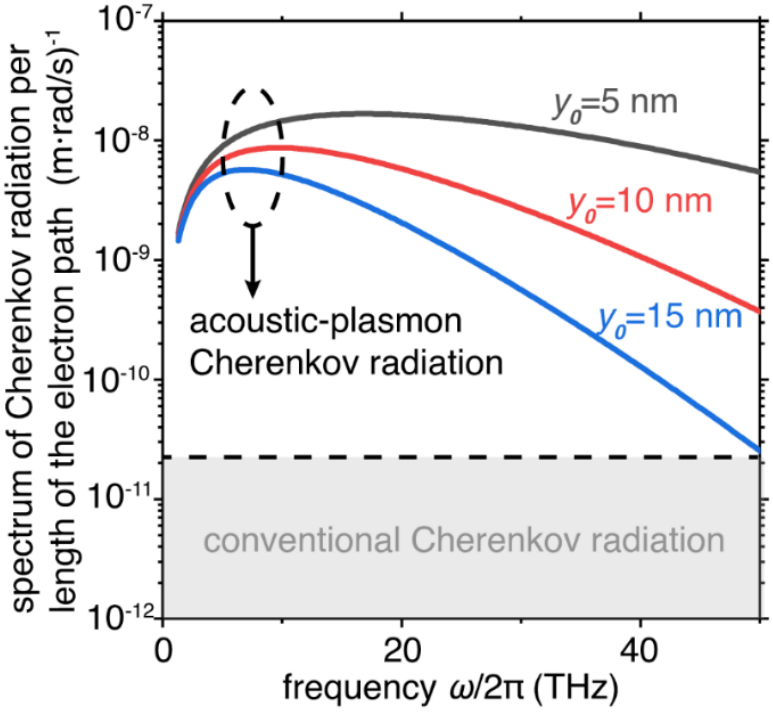

Figure S5. Influence of distance between the free electron and graphene sheet on the spectrum of acoustic-plasmon Cherenkov radiation. The black dashed line indicates the upper bound of the spectrum for conventional Cherenkov radiation induced by a swift electron moving inside any homogeneous dielectrics.

Section S8. More discussions on the angular spectrum of Cherenkov radiation

We plot in Figure S6 the relation between the emission frequency of surface-plasmon Cherenkov radiation emission angle. The emission angle is defined as the one between the propagation direction of surface plasmons and electron trajectory. Such an emission angle is generally dispersive as $\theta(\omega) = \cos^{-1}\left(v_{\mathrm{p}}(\omega)/v_{\mathrm{e}}\right)$. The upper bound of emission angle is fixed at $\theta_{\max} = \cos^{-1}(v_{\mathrm{F}}/v_{\mathrm{e}})$, while the lower bound of emission angle depends on the separation between the graphene sheet and metal substrate. From Figure S6, we find that when the separation *d*>4 nm, the minimum achievable emission angle is zero since $\min v_{\mathrm{p}} < v_{\mathrm{e}}$; when the separation *d*=2 nm, the minimum achievable emission angle is non-zero, i.e.,

$\theta_{\text{min}} = \cos^{-1}(v_{\text{p,min}}/v_{\text{e}})$ since $v_{\text{p,min}} > v_{\text{e}}$. Therefore, the acoustic-plasmon Cherenkov radiation is more directional than conventional surface-plasmon Cherenkov radiation.

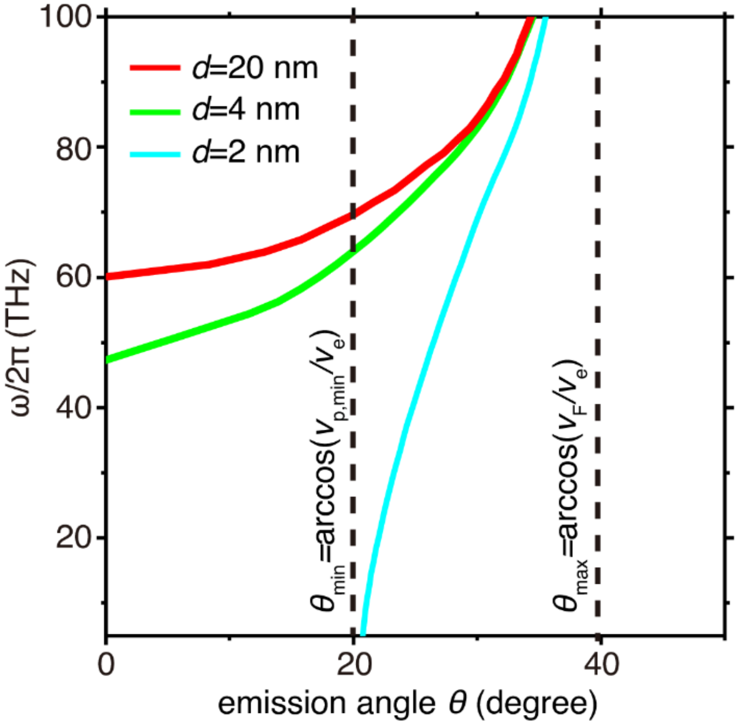

Figure S6. Relation between the emission frequency of surface-plasmon Cherenkov radiation and emission angle. The emission angle is defined as the one between the propagation direction of surface plasmons and electron trajectory.

We further demonstrate the influence of the separation distance, permittivity of dielectric spacer and graphene's chemical potential on the angular width of Cherenkov radiation in the time domain. From Figure S7, the decrease of separation distance will effectively reduce the angular width, making Cherenkov radiation more directional. Moreover, the permittivity of dielectric spacer and chemical potential in the graphene provide additional routes to control the directionality. As shown in Figure S8, the increase of

permittivity of dielectric spacer will enhance the directionality of acoustic-plasmon Cherenkov radiation while the increase of chemical potential will reduce the directionality.

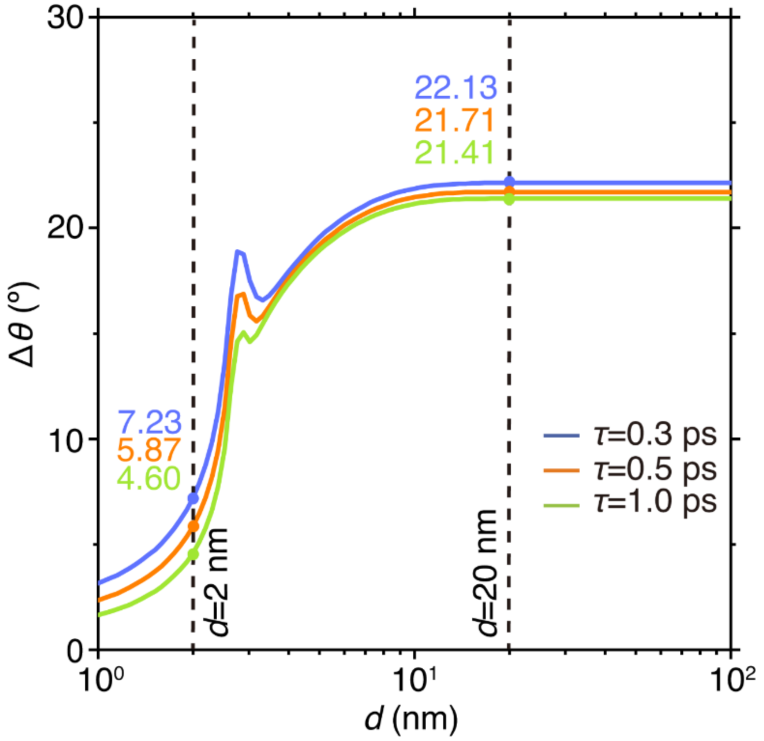

Figure S7. The angular width of Cherenkov radiation versus the separation distance. The studied relaxation time are $\tau = 0.3$ ps, $\tau = 0.5$ ps and $\tau = 1.0$ ps, respectively. Other parameter setups are: $d = 2$ nm, $v_e = 1.3v_F$, and $\mu_c = 0.3$ eV.

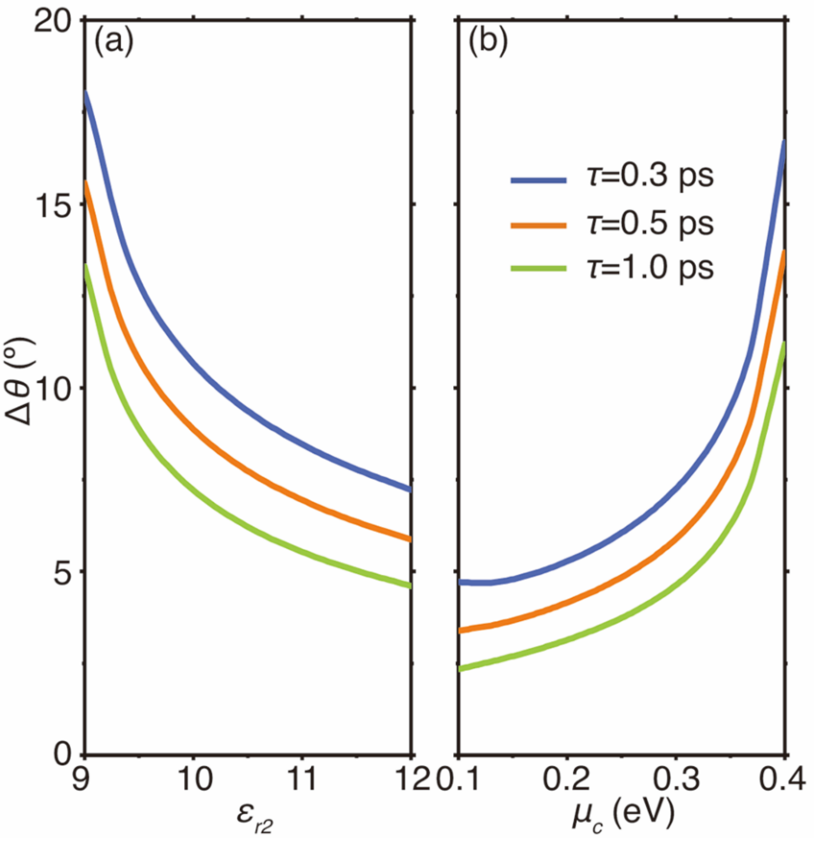

Figure S8. Controlling the angular width of acoustic-plasmon Cherenkov radiation. a) Influence of the permittivity of dielectric spacer on the angular width. The chemical potential is $\mu_{\mathrm{c}} = 0.3$ eV. b) Influence of graphene's chemical potential on the angular width. The permittivity of dielectric is $\varepsilon_{\mathrm{r2}} = 12$. In all the panels, the studied relaxation time are $\tau = 0.3$ ps, $\tau = 0.5$ ps and $\tau = 1.0$ ps, respectively. Other parameter setups are: $d = 2$ nm and $v_{\mathrm{e}} = 1.3v_{\mathrm{F}}$.

Section S9: Measuring nanoscale distance via sharp bandwidth transition of Cherenkov radiation

Here we reveal that the observed sharp bandwidth transition of Cherenkov radiation can inspire future research on the nanoscale distance sensing. The sharp bandwidth transition arises when conventional plasmons are converted to acoustic plasmons. We remark that such a sharp bandwidth transition is highly sensitive to the separation variations. As clearly demonstrated in Figure S9, the chemical potentials μ_{c} of

graphene that enable the sharp transition are 0.34, 0.28 and 0.24 eV, respectively, when the separation d is 2.5, 3.0 and 3.5 nm. Thus, our structure offers a new platform to measure the nanoscale distance.

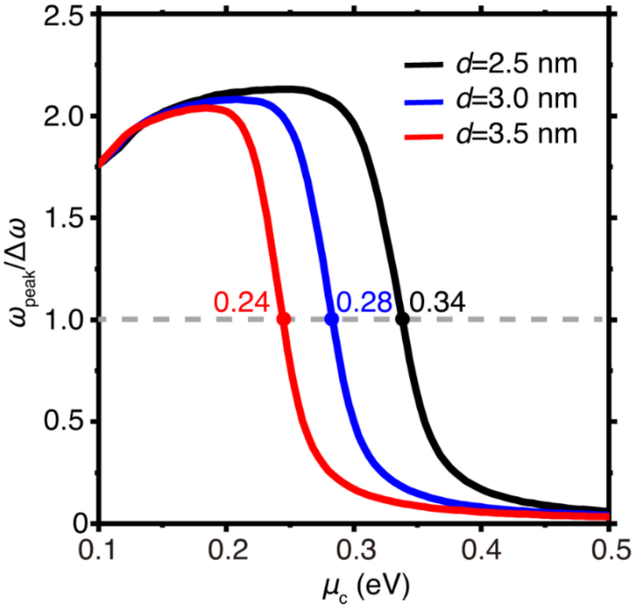

Figure S9. Normalized bandwidth of surface-plasmon Cherenkov radiation as the function of the chemical potential of graphene. Other adopted parameters are: $v_e = 1.3v_F$, $\tau = 0.5$ ps, and $\varepsilon_{r2} = 12$.

Section S10. Dispersionless-plasmon Cherenkov radiation on metal/semiconductor substrate

In this section, we reveal that the semiconductor/metal considering the nonlocality also provides a potential platform to realize the dispersionless plasmons, thus enabling the broadband enhancement of Cherenkov radiation. Here, we study the configuration as shown in the insets of Figure 5: a free electron (in region 1) is moving atop a semiconductor or metal substrate (region 2). When the semiconductor/metal is in the local approximation, the permittivity is given by the local Drude model $\varepsilon_{rT2} = \varepsilon_\infty - \frac{\omega_p^2}{\omega(\omega+i\gamma)}$. nonlocality arises if the quantum repulsion between induced surface charges at the semiconductor/metal interface is taken into consideration [6]. As a consequence, the longitudinal field is induced with the

corresponding permittivity given by the nonlocal Drude model $\varepsilon_{\mathrm{rL2}} = 1 - \frac{\omega_p^2}{\omega(\omega+\mathrm{i}\gamma)-(k_L\beta)^2} = 0$, where k_L is the wavevector of longitudinal field and $\beta = \sqrt{3/5}\,v_{\mathrm{F}}$ is the nonlocal parameter.

To determine the dispersion relation of surface plasmons with the consideration of nonlocality, we add the longitudinal field induced by the nonlocality (in addition to the transverse field) in the semiconductor/metal region (region 2). The expression of longitudinal field takes the form as

$$E_\rho(\bar{r},\omega) = \mathrm{i}qT_{p2}^l e^{-\mathrm{i}k_{y2}^l y} e^{\mathrm{i}q\rho} \tag{10.1a}$$

$$E_y(\bar{r},\omega) = -\mathrm{i}k_{y2}^l T_{p2}^l e^{-\mathrm{i}k_{y2}^l y} e^{\mathrm{i}q\rho} \tag{10.1b}$$

$$H_h(\bar{r},\omega) = 0 \tag{10.1c}$$

where $k_{y2}^l = \sqrt{k_L^2 - q^2}$ is the y-component of wavevector of longitudinal field [9]. By applying the traditional boundary condition (i.e., the tangent continuity of field component) and additional boundary condition (i.e, the normal continuity of current density) [6], we can determine the dispersion relation of surface plasmons in nonlocal case expressed as

$$\frac{k_{y1}}{\varepsilon_{\mathrm{r1}}} + \frac{k_{y2}}{\varepsilon_{\mathrm{rT2}}} - \left(\frac{1}{\varepsilon_\infty} - \frac{1}{\varepsilon_{\mathrm{rT2}}}\right)\frac{q^2}{k_{y2}^l} = 0. \tag{10.2}$$

Here $\varepsilon_{\mathrm{r1}} = 1$ if the adopted dielectric in the region 1 is air. Particularly, if $q \to 0$, dispersion relation Eq. (10.2) is reduced to the local one.

With a similar methodology, we can obtain the reflection coefficients from the interface of semiconductor/metal as

$$R_{\mathrm{TM}} = -\frac{\frac{k_{y1}}{\varepsilon_{\mathrm{r1}}} - \frac{k_{y2}}{\varepsilon_{\mathrm{rT2}}} + \left(\frac{1}{\varepsilon_\infty} - \frac{1}{\varepsilon_{\mathrm{rT2}}}\right)\frac{q^2}{k_{y2}^l}}{\frac{k_{y1}}{\varepsilon_{\mathrm{r1}}} + \frac{k_{y2}}{\varepsilon_{\mathrm{rT2}}} - \left(\frac{1}{\varepsilon_\infty} - \frac{1}{\varepsilon_{\mathrm{rT2}}}\right)\frac{q^2}{k_{y2}^l}} \tag{10.3}$$

Making use of the reflection coefficients Eq. (10.3) and formula Eq. (4.6), one can compute the spectrum of surface-plasmon Cherenkov radiation with the consideration.

As shown in Figure S10a, when the optical response of semiconductor InSb is in the local approximation (without the consideration of nonlocality), the plasmonic frequency is always smaller than 2.8 THz. However, if we consider the nonlocality, such a maximum plasmonic frequency disappears. In fact, the plasmonic frequency approximately satisfies $\omega \propto q$ if $q/(\omega/c)$ is greater than 200. In other words, the surface plasmons on semiconductors are dispersionless plasmons in the deep subwavelength regime. Similar phenomenon is also observed in Figure S10b, where the substrate InSb is replaced by the metal Ag.

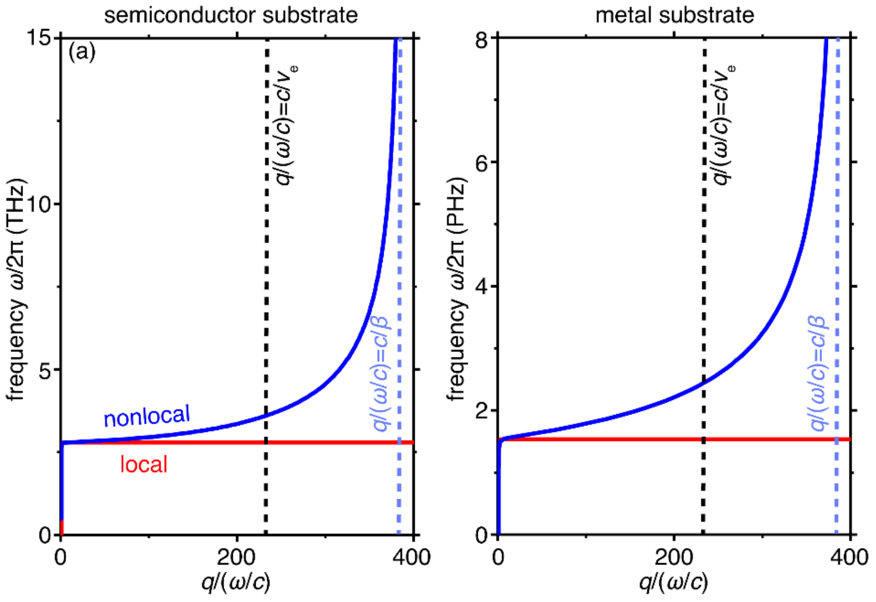

Figure S10. Dispersion relation of surface plasmons. a) Dispersion relation of surface plasmons at the interface of semiconductor substrate. In calculation, we choose InSb as the semiconductor. Parameters adopted for Indium antimonide (InSb) are: the permittivity for bound charges $\varepsilon_{\infty} = 15.6$, the plasmonic frequency $\omega_p/2\pi = 7.16 \times 10^{13}$ THz and the damping rate $\gamma = 0.01\omega_p$. b) Dispersion relation of surface plasmons at the interface of metal substrate. In calculation, we choose silver (Ag) as the metal. Parameters adopted for the Ag are: the permittivity for bound charges $\varepsilon_{\infty} = 1$, the plasmonic frequency $\omega_p = 1.37 \times 10^{16}$ rad/s and the damping rate $\gamma = 0.01\omega_p$. Here, $v_e = 1.3v_F$ and $v_F = c/300$.

Section S11. Detection scheme of acoustic-plasmon Cherenkov radiation

The detection of acoustic-plasmon Cherenkov radiation can be achieved with the silicon-based blocked impurity band (Si-based BIB) detectors and grating technology (Figure S11). To be specific, the acoustic surface plasmons generated from the free electrons will interact with the sub-wavelength grating coupler, leading to the free-space photons in midinfrared to terahertz frequencies. The free-space photons in midinfrared to terahertz frequencies are probed by the Si-based BIB detector [10,11].

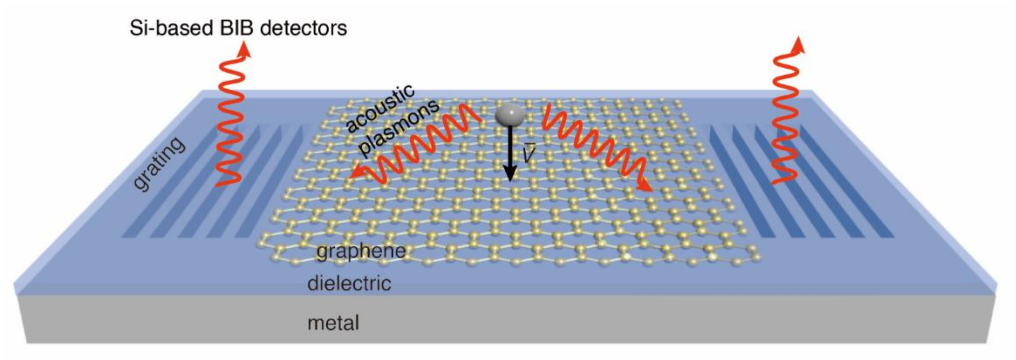

Figure S11. Scheme for the detection of acoustic-plasmon Cherenkov radiation. The grating coupler is etched on the metallic substrate. The acoustic surface plasmons generated from the free electrons is coupled to the free-space photons through the sub-wavelength grating coupler. Si-based BIB detector is applied to detect free-space photons in midinfrared to terahertz frequencies.

Reference:

[1] F. H. Koppens, D. E. Chang, and F. J. Garcia de Abajo, *Nano Lett.* **2011**, 11, 3370.
[2] X. Lin *et al.*, *Proc. Natl. Acad. Sci.* **2017**, 114, 6717.
[3] M. J. Lighthill and J. Lighthill, *Waves in fluids*, Cambridge university press, **2001**.
[4] X. Lin *et al.*, *Sci. Adv.* **2017**, 3, e1601192.
[5] X. Shi, X. Lin, F. Gao, H. Xu, Z. Yang, and B. Zhang, *Phys. Rev. B.* **2015**, 92, 081404.
[6] H. Hu, X. Lin, J. Zhang, D. Liu, P. Genevet, B. Zhang, and Y. Luo, *Laser Photonics Rev.* **2020**, 14, 2000149.
[7] N. Rivera and I. Kaminer, *Nat. Phys.* **2020**, 2, 538.
[8] R. Dahan *et al.*, *Nat. Phys.* **2020**, 16, 1123.

[9] Y. Luo, A. Fernandez-Dominguez, A. Wiener, S. A. Maier, and J. Pendry, *Phys. Rev. Lett.* **2013**, 111, 093901.
[10] A. Rogalski and F. Sizov, *Opto-electron. Rev.* **2011**, 19, 346.
[11] H. Zhu, Z. Weng, J. Zhu, H. Wu, N. Li, and N. Dai, *IEEE Trans. Electron. Devices*. **2017**, 64, 1094.
